# High-glucose-induced miR-214-3p inhibits BMSCs osteogenic differentiation in type 1 diabetes mellitus

**DOI:** 10.1038/s41420-019-0223-1

**Published:** 2019-11-12

**Authors:** Rongze Wang, Yuanxu Zhang, Fujun Jin, Gongchen Li, Yao Sun, Xiaogang Wang

**Affiliations:** 1 Beijing Advanced Innovation Center for Big Data-Based Precision Medicine, School of Biological Science and Medical Engineering, Beihang University, Beijing, China; 2 Institute of Biomedicine, College of Life Science and Technology, Jinan University, Guangzhou, China; 3 Integrated Chinese and Western Medicine Postdoctoral Research Station, Jinan University, Guangzhou, China; 4 Department of Oral Implantology, School of Stomatology, Tongji University, Shanghai Engineering Research Center of Tooth Restoration and Regeneration, Shanghai, China; 5 Clinical Research Platform for Interdiscipline of Stomatology, The First Affiliated Hospital of Jinan University, Jinan University, Guangzhou, China

**Keywords:** RNA, Diseases, Differentiation

## Abstract

Type 1 diabetes mellitus (T1DM) is an autoimmune insulin-dependent disease associated with destructive bone homeostasis. Accumulating evidence has proven that miRNAs are widely involved in the regulation of bone homeostasis. However, whether miRNAs also regulate osteogenic differentiation of bone marrow mesenchymal stem cells (BMSCs) in T1DM mice is under exploration. In this study, miRNA microarray was utilized to screen the differentially expressed miRNAs, which uncovered that miR-214-3p potentially inhibited BMSCs osteogenic differentiation in T1DM mice. We found that high glucose suppressed BMSCs osteogenic differentiation with significant elevation of the miR-214-3p expression. Further study found that the osteogenic differentiation of BMSCs was inhibited by AgomiR-214-3p while enhanced by AntagomiR-214-3p in BMSCs supplemented with high glucose. Moreover, we found that miR-214-3p knockout T1DM mice were resistant to high-glucose-induced bone loss. These results provide a novel insight into an inhibitory role of high-glucose-induced miR-214-3p in BMSCs osteogenic differentiation both in vitro and in vivo. Molecular studies revealed that miR-214-3p inhibits BMSCs osteogenic differentiation by targeting the 3′-UTR of β-catenin, which was further corroborated in human bone specimens and BMSCs of T1DM patients. Taken together, our study discovered that miR-214-3p is a pivotal regulator of BMSCs osteogenic differentiation in T1DM mice. Our findings also suggest that miR-214-3p could be a potential target in the treatment of bone disorders in patients with T1DM.

## Introduction

Type 1 diabetes mellitus (T1DM) is a chronic metabolic disorder resulting from hypoinsulinemia due to the destruction of the pancreatic β-cells^1^. T1DM is commonly diagnosed in childhood and young adults and accounts for approximately 10% of all diabetes cases^2,3^. Patients with T1DM have increased risk of bone complications such as osteoporosis, bone fracture and impaired bone healing^4–6^. Bone marrow mesenchymal stem cells (BMSCs) are a major source of osteoblasts and are crucial for bone remodeling and regeneration^7,8^. Previous studies have revealed that diabetes have direct detrimental effects on BMSCs, which in turn are responsible for diabetic osteopenia^9–11^, suggesting that BMSCs could be a potential target in the treatment of T1DM-associated bone complications.

MicroRNAs (miRNAs) are small noncoding RNAs with 19–25 nucleotides in length^12^. They mainly function as negative regulators by binding to the 3′-untranslated region (3′-UTR) of their target mRNAs, leading to mRNA degradation or translation inhibition^13,14^. Emerging evidence shows that miRNAs are widely involved in the process of osteogenic differentiation and bone formation; meanwhile dysregulation of these miRNAs has been associated with bone disorders^15–17^. Furthermore, differentially expressed pattern of miRNAs has been shown to involve in the process of bone formation in T2DM. For instance, Gong et al. have found that miR-132 is significantly upregulated in a mouse model of T2DM, which suppresses osteogenic differentiation through downregulating Sirt1^18^. Congruently, Li et al. reported that let-7c-2-3p, let7a-1-3p, and miR-322-3p were differently expressed under T2DM conditions, which target and block the expression of Wnt/β-catenin pathway genes^19^. However, whether altered expression of miRNAs in T1DM has any effects on BMSCs osteogenic differentiation is still largely unknown, despite its pharmacological significances. We thus aimed to identify differentially expressed miRNAs in T1DM mice and further explored the mechanistic role of these miRNAs involved in regulating the BMSCs osteogenic differentiation.

## Results

### Upregulation of miR-214-3p in bone tissue of diabetic mice

**Fig. 1:**
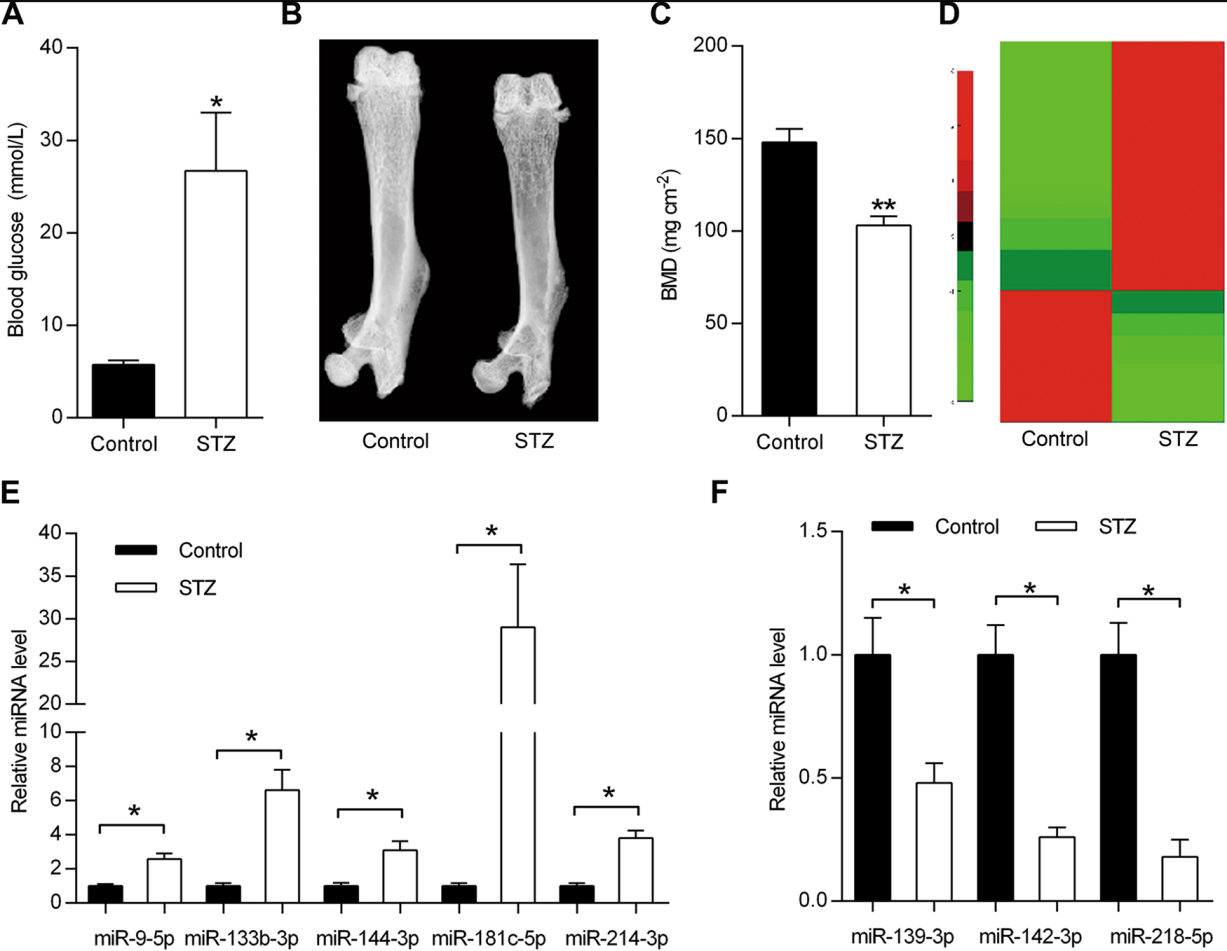
Identification of differentially expressed miRNAs in bone tissues from WT and diabetic mice. **a** The measurement of blood glucose concentrations in STZ-induced diabetic mice and control WT mice (*n* = 5). **b** Representative dual-energy X-ray images of mouse femur from STZ-induced diabetic mice or control mice. **c** Bone mineral density (BMD) of STZ-induced diabetic mice or control mice (*n* = 5). **d** Heatmap of differentially expressed miRNAs of bone specimens from STZ-induced diabetic mice or normal mice. **e**, **f** qRT-PCR analysis of miRNA expression in bone specimens from STZ-induced diabetic mice or control mice (*n* = 3). All data are expressed as mean ± SEM, *p < 0.05, **p < 0.01

To explore the mechanistic role of miRNAs in T1DM-induced bone formation defects, we first established an STZ-induced T1DM mouse model by a high-dose injection of STZ as previously described^20^. The blood glucose concentration of T1DM mice was significantly upregulated in diabetic mice, relative to the control mice (Fig. 1a), indicating the successful construction of T1DM model. Representative dual-energy X-ray images showed that bone mass in the femurs of STZ-induced T1DM mice was lower than control mice (Fig. 1b), which was further corroborated by the reduction of the BMD quantification (Fig. 1c). Consistent with the previous reports, our results clearly showed that the bone mass was greatly decreased in T1DM mice. These data together indicate that we successfully constructed the T1DM mouse model. Then the differentially expressed miRNAs were identified in bone tissues of T1DM mice by miRNA microarray assay (Fig. 1d), and qRT-PCR analysis demonstrated the elevated expression levels of miR-9-5p, miR-133b-3p, miR-144-3p, miR-181c-5p, miR-214-3p in diabetic bone tissues, and the decreased expression levels of miR-139-3p, miR-142-3p, miR-218-5p (Fig. 1e, f). Given the established role of miR-214-3p in inhibiting osteoblast differentiation and bone formation^21^, we further focused on miR-214-3p for further study.

### Blockage of diabetes-induced bone loss in miR-214-3p knockout mice

**Fig. 2:**
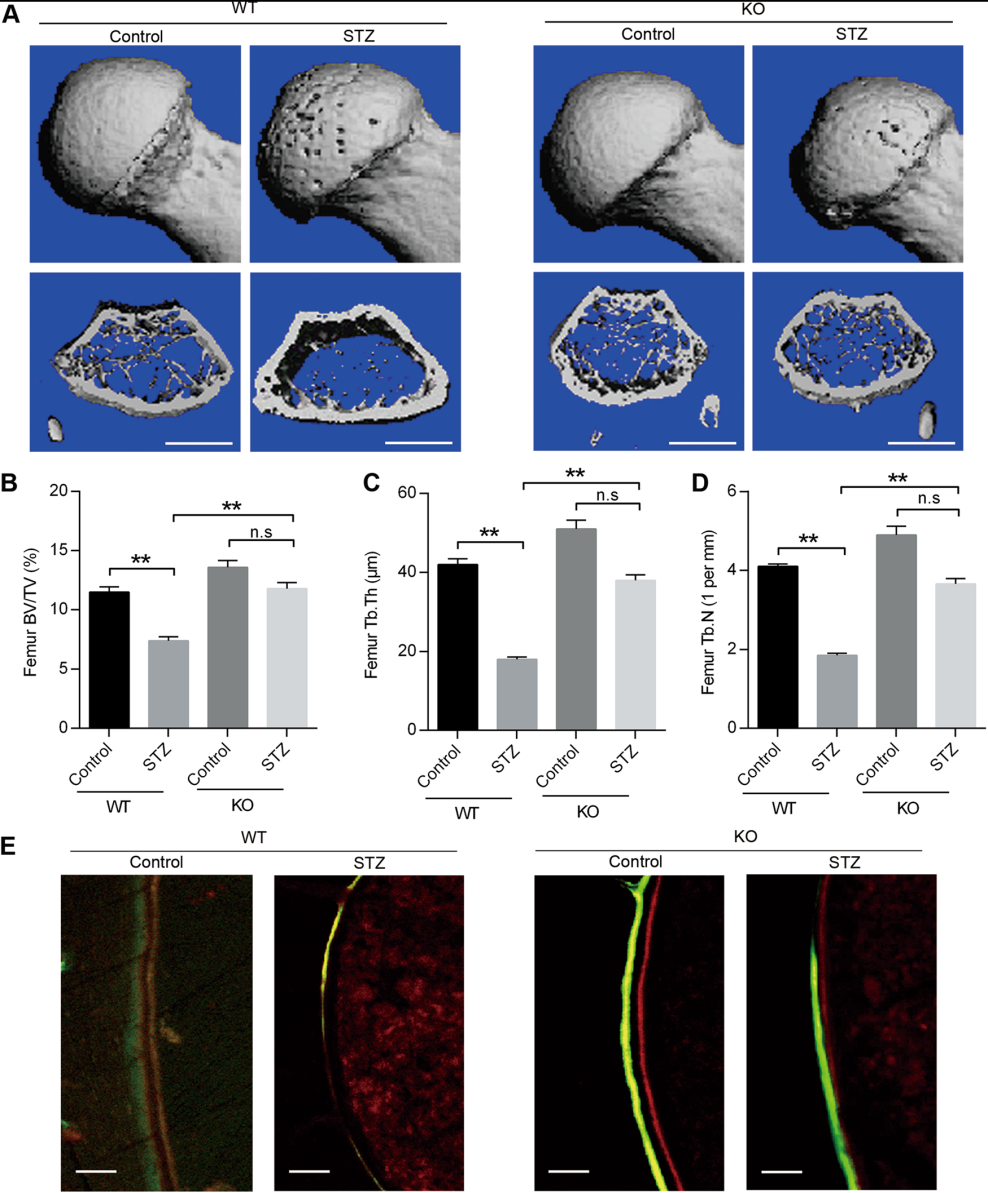
Reversal of diabetes-induced bone loss in miR-214-3p knockout mice. **a** Representative micro-CT images of distal femur from WT and miR-214-3p knockout mice treated with or without STZ. Quantitative analysis of μCT parameters, including **b** bone volume per total volume; BV/TV, **c** trabecular thickness; Tb.Th, **d** trabecular number; Tb.N. Scale bars, 1 mm. **e** Representative images of new bone formation assessed by Red-calcein double labeling in WT and miR-214-3p knockout mice treated with or without STZ. Scale bars, 10 μm. All data are expressed as mean ± SEM, *n* = 6, **p < 0.01

We next sought to determine the regulatory role of miR-214-3p in T1DM-induced bone loss in vivo, by establishing a model of T1DM in miR-214-3p knockout mice. The results showed that STZ treatment induced a significant bone loss in WT mice compared with vehicle-treated WT mice. However, the bone mass was comparable in STZ-treated miR-214-3p knockout mice than vehicle-treated WT mice (Fig. 2a). Similarly, quantitative analysis of femur histomorphological parameters showed that the deletion of miR-214-3p could restore bone volume fraction (BV/TV), trabecular thickness (Tb.Th) and trabecular number (Tb.N) (Fig. 2b–d). To further confirm the effects of miR-214-3p on bone formation, double labeling assay was performed to measure the bone-formation rate (BFR), which showed that mice in the STZ-treated WT mice exhibited significantly lower BFR levels than the control mice while there was no difference between miR-214-3p knockout mice treated with or without STZ (Fig. 2e). These data demonstrated the crucial role of miR-214-3p in bone formation in T1DM mice.

### High glucose induces miR-214-3p expression in BMSCs

**Fig. 3:**
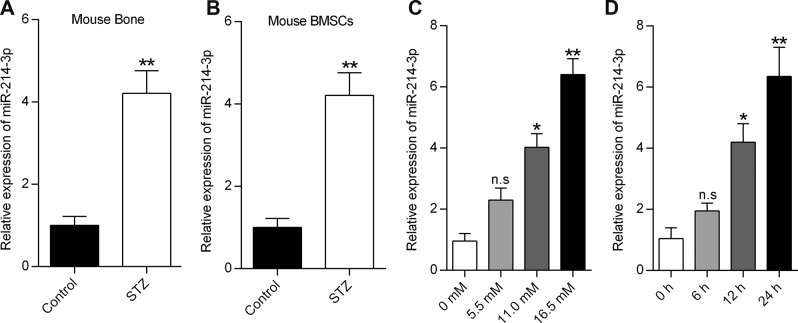
High glucose induces miR-214-3p expression in BMSCs. **a** qRT-PCR analysis of miR-214-3p levels in bone specimens from mice treated with or without STZ (*n* = 3). **b** qRT-PCR analysis of miR-214-3p levels in BMSCs extracted from the mice treated with or without STZ (*n* = 3). **c** qRT-PCR analysis of miR-214-3p levels in BMSCs treated with glucose at different concentrations for 24 h (*n* = 3). **d** qRT-PCR analysis of miR-214-3p expression in BMSCs at the indicated time points after treatment with glucose at 16.5 mM (*n* = 3). All data are expressed as mean ± SEM, *p < 0.05, **p < 0.01

We further sought to test whether high glucose was responsible for the elevated expression of miR-214-3p in T1DM. To this end, we compared the expression levels of miR-214-3p in bone specimens from STZ-induced diabetic mice and corresponding control mice. A significant increase of miR-214-3p levels was observed in both bone specimens and isolated BMSCs from the STZ-induced diabetic mice, relative to the control mice (Fig. 3a, b). These results strongly indicated that glucose could induce the expression of miR-214-3p. To further verify this hypothesis, we investigated the link between high glucose and miR-214-3p expression, by treating BMSCs with different doses of glucose for 24 h. Subsequently, the qRT-PCR analysis showed that glucose treatment enhanced the miR-214-3p expression in dose- and time-dependent manner (Fig. 3c, d). Taken together, these data suggest that high glucose induces the miR-214-3p expression in BMSCs both in vitro and in vivo.

### High glucose-induced miR-214-3p inhibits BMSCs osteogenic differentiation

**Fig. 4:**
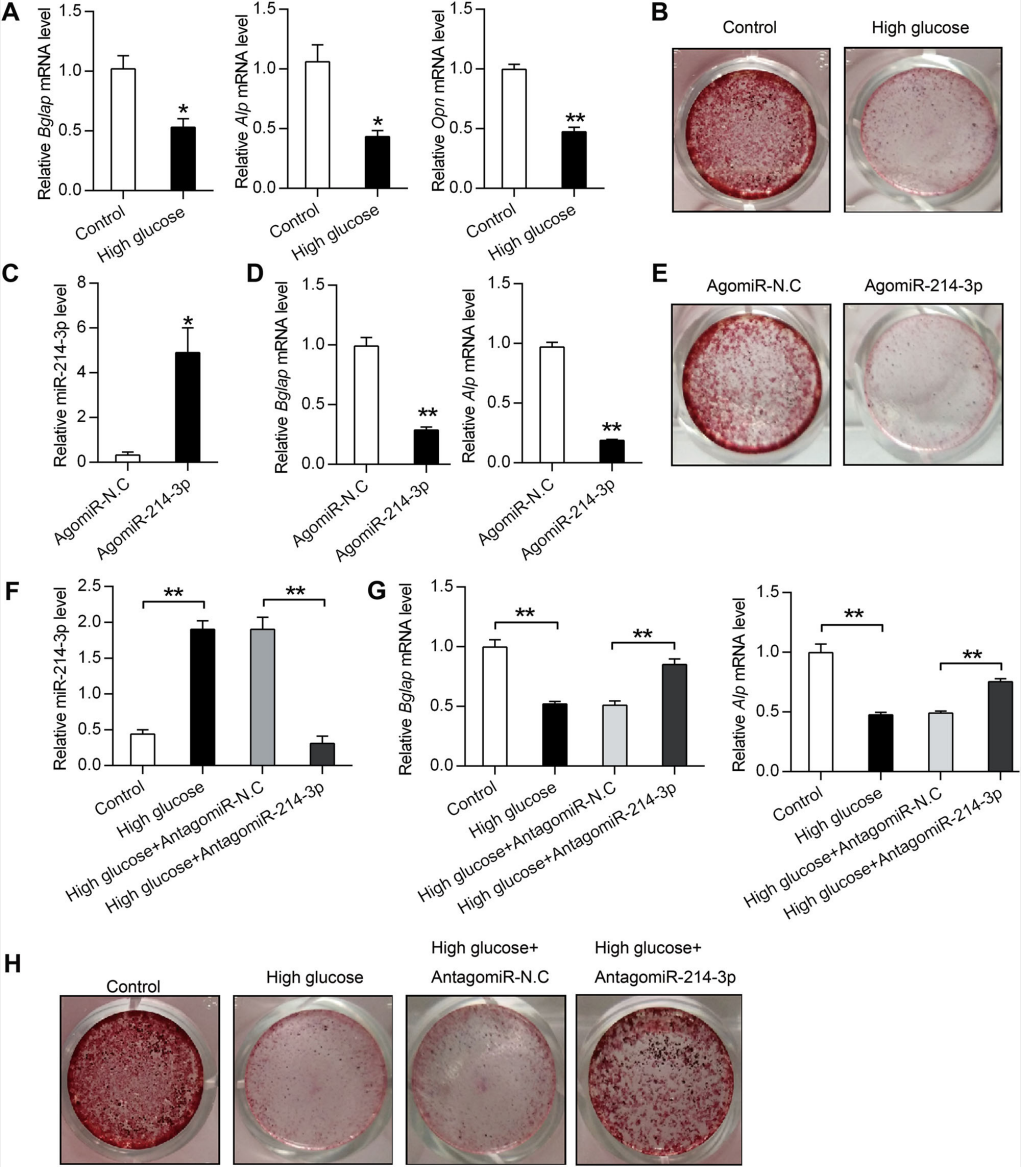
High-glucose-induced miR-214-3p inhibits BMSCs osteogenic differentiation. **a** qRT-PCR analysis of *Bglap*, *Alp*, *Opn* in BMSCs after treatment with high glucose for 3 weeks (*n* = 3). **b** Alizarin red staining of calcium deposition in BMSCs after treatment with high glucose for 3 weeks. **c**, **d** qRT-PCR analysis of miR-214-3p, *Bglap* and *Alp* mRNA levels in BMSCs after treatment with 200 μM AgomiR-214-3p or AgomiR-N.C in osteogenic medium for 3 weeks (*n* = 3). **e** Alizarin red staining of calcium deposition in BMSCs after treatment with 200 μM AgomiR-214-3p or AgomiR-N.C in osteogenic medium for 3 weeks. **f**, **g** qRT-PCR analysis of miR-214-3p, *Bglap* and *Alp* mRNA levels in BMSCs after treatment with high glucose together with or without 200 μM AntagomiR-214-3p and AntagomiR-N.C for 3 weeks (*n* = 3). **h** Alizarin red staining of BMSCs after treatment with high glucose together with 200 μM AntagomiR-214-3p or AntagomiR-N.C for 3 weeks. All data are expressed as mean ± SEM, *p < 0.05, **p < 0.01

Considered that miR-214-3p was a well-known negative regulator of osteogenic differentiation^21,22^, and that high glucose induced miR-214-3p expression in BMSCs, we tested the effect of high glucose on osteogenic differentiation. The results showed that high glucose induced osteogenic defects (Fig. 4a, b). To further confirm whether miR-214-3p was involved in high-glucose-induced osteogenic defects, we treated BMSCs with AgomiR-214-3p (an miR-214-3p agonist) or AgomiR-N.C. The results showed that AgomiR-214-3p treatment greatly increased intracellular miR-214-3p expression levels (Fig. 4c). Besides, qRT-PCR analysis of osteogenic marker genes showed miR-214-3p significantly attenuated *Bglap* and *Alp* expression (Fig. 4d). Moreover, Alizarin Red staining showed that BMSCs treated with miR-214-3p agonist displayed decreased calcium deposition and the areas of mineralized matrices, indicating reduced osteogenic differentiation of BMSCs (Fig. 4e). Next, we treated BMSCs with high glucose to simulate the endogenous expression of miR-214-3p and utilized AntagomiR-214-3p (an miR-214-3p inhibitor) to further demonstrate the role of high-glucose-induced miR-214-3p in BMSCs osteogenic differentiation. The results revealed that BMSCs in the high glucose group displayed higher mRNA levels of miR-214-3p while AntagomiR-214-3p decreased miR-214-3p expression induced by high glucose (Fig. 4f). Moreover, the mRNA levels of *Bglap* and *Alp* were also analyzed. Results showed that AntagomiR-214-3p could recover the reduced expression of *Bglap* and *Alp* which was induced by high glucose stimuli (Fig. 4g). Furthermore, Alizarin Red staining showed that BMSCs treated with high glucose exerted reduced number of mineralized nodules and mineralized matrix accumulation while AntagomiR-214-3p significantly attenuated the inhibitory effects of high glucose treatment on osteogenesis differentiation (Fig. 4h). Taken together, our results suggest that miR-214-3p contributes to high-glucose-induced BMSCs osteogenic defects.

### High-glucose-induced miR-214-3p functionally targets β-catenin

**Fig. 5:**
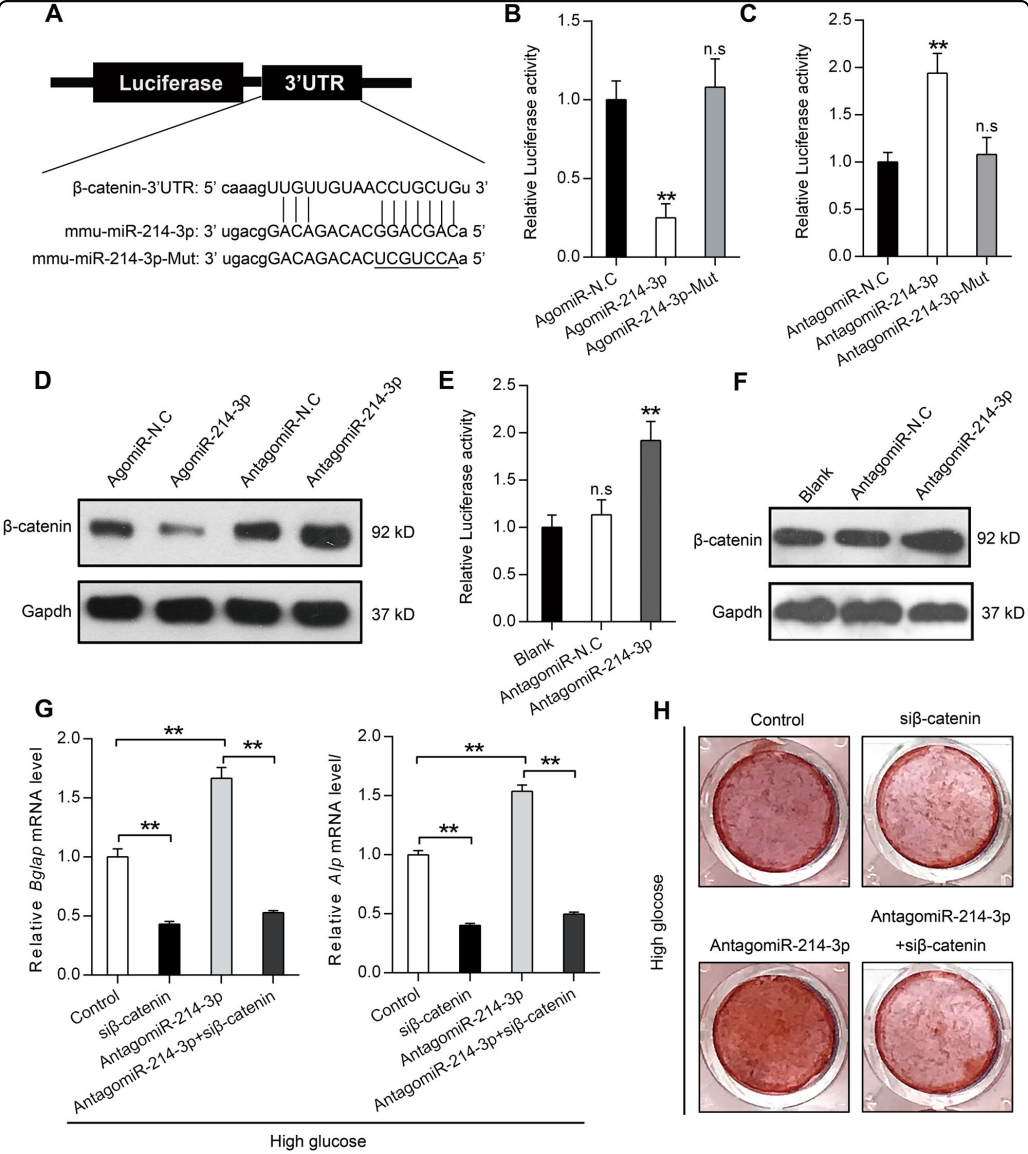
High-glucose-induced miR-214-3p functionally targets β-catenin. **a** Schematic diagram illustrating the design of luciferase reporters with the β-catenin 3′-UTR. The sequences of miR-214-3p and miR-214-3p-Mut are also shown. **b** The effect of AgomiR-214-3p, AgomiR-N.C and AgomiR-214-3p-Mut on luciferase activity in BMSCs after transfection with the β-catenin 3′-UTR reporter (*n* = 5). **c** The effect of AntagomiR-214-3p, AntagomiR-N.C and AntagomiR-214-3p-Mut on luciferase activity in BMSCs after transfection with the β-catenin 3′-UTR reporter (*n* = 5). **d** Western blot analysis of β-catenin protein levels in BMSCs after treatment with AgomiR-214-3p, AntagomiR-214-3p and their corresponding negative controls. **e** The effect of AntagomiR-214-3p and AntagomiR-N.C on luciferase activity in BMSCs supplemented with high glucose (*n* = 5). **f** Western blot analysis of β-catenin protein levels in BMSCs supplemented with high glucose after treatment with AntagomiR-214-3p or AntagomiR-N.C. **g** qRT-PCR analysis of *Bglap* and *Alp* in BMSCs after treatment with β-catenin siRNA and AntagomiR-214-3p in osteogenic medium supplemented with high glucose (*n* = 3). **h** Alizarin red staining of calcium deposition in BMSCs after treatment with β-catenin siRNA and AntagomiR-214-3p in osteogenic medium supplemented with high glucose. All data are expressed as mean ± SEM, **p < 0.01

Reasoning the above speculated functional significance of miR-214-3p, we next predicted the potential target of miR-214-3p by using PITA, miRanda and miRDB. The results revealed that there was a potential binding site in the 3′-UTR of β-catenin for the miR-214-3p sequence (Fig. 5a). To validate the interaction between miR-214-3p and its putative target gene β-catenin, we constructed a luciferase reporter vector carrying the 3′-UTR of β-catenin (Fig. 5a). The BMSCs were treated with AgomiR-214-3p, AgomiR-214-3p-Mut or AgomiR-214-3p-N.C along with the transfection of luciferase reporter vector. The results showed that AgomiR-214-3p, but not AgomiR-214-3p-Mut significantly reduced the luciferase activity (Fig. 5b). By contrast, we found that AntagomiR-214-3p resulted in the significant increase of luciferase activity while AntagomiR-214-3p-Mut had no effects on luciferase activity (Fig. 5c), which showed that miR-214-3p could bind to the 3′-UTR of β-catenin. To further experimentally validate that β-catenin expression was regulated by miR-214-3p, we treated BMSCs cells with AgomiR-214-3p or AntagomiR-214-3p and detected the protein levels of β-catenin by Western blot analysis, which revealed that AgomiR-214-3p significantly repressed the levels of endogenous β-catenin while AntagomiR-214-3p upregulated the protein level of β-catenin (Fig. 5d). To investigate the effect of high-glucose-induced endogenous miR-214-3p on β-catenin, we performed luciferase assay in BMSCs supplemented with high glucose. Results showed that AntagomiR-214-3p increased luciferase activity (Fig. 5e). Besides, increased β-catenin protein expression was also observed in cells treated with AntagomiR-214-3p (Fig. 5f). Next, to explore whether β-catenin does contribute to miR-214-3p-induced osteogenic defects, we transfected BMSCs with β-catenin siRNA alone or combined with AntagomiR-214-3p under the high glucose conditions. The results of qRT-PCR analysis showed that β-catenin silence could decrease the expression of *Bglap* and *Alp*, which was elevated by AntagomiR-214-3p treatment (Fig. 5g). Consistently, Alizarin Red staining also showed that AntagomiR-214-3p could enhance the osteogenic differentiation of BMSCs under high glucose conditions while β-catenin silence could abolish this effect (Fig. 5h). Taken together, our results confirm that β-catenin is a direct target gene of miR-214-3p and that β-catenin expression is suppressed by miR-214-3p in BMSCs during high glucose treatment, thus contributing to the inhibition of osteogenic differentiation.

### miR-214-3p and β-catenin expression in diabetic osteoporosis patients

**Fig. 6:**
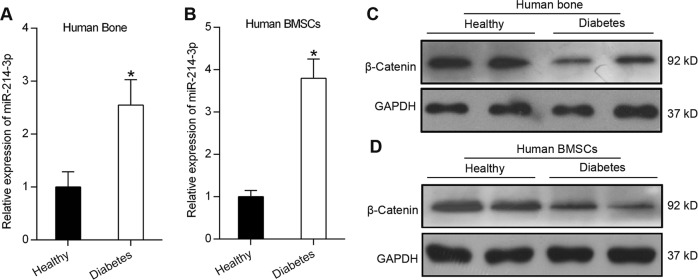
miR-214-3p and β-catenin expression in diabetic osteoporosis patients. **a** qRT-PCR analysis of miR-214-3p mRNA levels in human bone specimens from healthy people or patients with T1DM (*n* = 3). **b** qRT-PCR analysis of miR-214-3p mRNA levels in BMSCs from healthy people or patients with T1DM (*n* = 3). **c** Western blot analysis of β-catenin in the bone specimens isolated from patients with T1DM or healthy people. **d** Western blot analysis of β-catenin in the BMSCs isolated from patients with T1DM or healthy people. All data are expressed as mean ± SEM, *p < 0.05

In order to examine the expression pattern of miR-214-3p and β-catenin in diabetic patients, we collected bone specimens from patients with the T1DM osteoporosis. qRT-PCR analysis revealed that the expression level of miR-214-3p was higher in bone specimens from T1DM osteoporosis patients compared to those from healthy individuals (Fig. 6a). Similarly, increased miR-214-3p expression was also observed in BMSCs isolated from T1DM patients (Fig. 6b). Western blot analysis showed that the protein level of β-catenin was decreased both in BMSCs and bone specimens of T1DM osteoporosis patients (Fig. 6c, d). The increased expression of miR-214-3p and decreased expression of β-catenin were consistent with previous results observed in T1DM mice.

## Discussion

T1DM is a prevalent systemic disease characterized by insulin insufficiency due to the destruction of the pancreatic beta cells^1^. It has been reported that T1DM has detrimental effects on bone remodeling, leading to a series of bone complications^23–25^. BMSCs, which possesses the capability to differentiate into osteoblasts, play an important role in bone remodeling^9,26,27^. Moreover, impaired osteogenic potential of BMSCs has been found in STZ-induced diabetic mice and may be responsible for T1DM-related bone complications^28^. Accordingly, a better understanding of the molecular mechanisms underlying the inhibition of BMSCs osteogenic differentiation may help to develop novel strategies to alleviate complications. There is copious evidence that miRNAs play an important role in the process of osteogenic differentiation^29–31^. Hence, we focus our investigations on the interaction between differentially expressed miRNAs and BMSCs osteogenic differentiation in T1DM mice.

Here, we identified several differentially expressed miRNAs in STZ-induced T1DM model and given the interesting expression pattern of miR-214-3p and its established regulatory role in diabetes and bone formation, miR-214-3p has garnered our research attention. miR-214-3p has been shown to play a crucial role in the regulation of hepatic gluconeogenesis^32^. miR-214-3p has also been reported to target Osterix, thereby suppressing osteogenic differentiation of C2C12 myoblast cells^33^. Besides, miR-214-3p was also reported to delay fracture healing in rats with osteoporotic fracture by inhibiting the BMP/Smad signaling pathway^34^. Given our previous study that miR-214-3p suppressed bone formation via targeting ATF4^21^, we hypothesized that the mechanistic role of miRNA might underpin bone loss in T1DM mice. To explore the mechanistic role of miR-214-3p in bone homeostasis in T1DM mice, we generated miR-214-3p knockout mice and conducted T1DM model. The miR-214-3p knockout mice exhibited improved trabecular architecture and increased bone mass in an STZ-induced T1DM model, indicating the crucial role of miR-214-3p in bone disorders associated with T1DM.

Hyperglycemia is a well-known hallmark of T1DM, which is also associated with a series of bone complications^35,36^. Besides, various previous studies have shown that high glucose concentrations serve as a crucial regulator for miRNA expression in diabetes^37,38^. Here, we report that miR-214-3p is overexpressed in BMSCs and bone specimens of STZ-induced diabetic mice. Besides, high glucose elevated the miR-214-3p expression in a time- and dose-dependent manner in BMSCs. Furthermore, AgomiR-214-3p reduced osteogenic gene expression and mineralized matrix accumulation in BMSCs, whereas AntagomiR-214-3p decreased the inhibition of BMSCs osteogenic differentiation induced by high glucose stimuli. The results indicate that high-glucose-induced miR-214-3p is responsible for the inhibition of BMSCs osteogenic differentiation.

As miRNAs control gene expression post-transcriptionally by binding to the 3′-UTR of their target mRNAs^39,40^, we utilized PITA, miRanda, and miRDB to predict the target gene of miR-214-3p and found a potential binding site in the 3′-UTR of β-catenin. β-catenin, a vital component of Wnt pathway, has been found to participate in the regulation of bone mass through regulating multiple aspects including osteoblast differentiation and function^41,42^. Previous study had also shown that the expression levels of β-catenin mRNAs and proteins were affected by miR-214-3p in a fracture healing model^43^. Consistently, our results confirmed that high-glucose-induced miR-214-3p reduced the mRNA and protein levels of β-catenin by binding to the 3′-UTR of β-catenin, thus contributing to the inhibition of osteogenic differentiation. Furthermore, miR-214-3p turned out to be upregulated in the diabetic osteoporosis patients compared with healthy people while the protein levels of β-catenin was decreased in diabetic osteoporosis patients.

In summary, our study demonstrated that high-glucose-induced miR-214-3p inhibited BMSCs osteogenic differentiation in T1DM mice and miR-214-3p knockout blocked high-glucose-induced bone formation defects in vivo. Collectively, these data uncovered the potential mechanistic role of miR-214-3p and its molecular target and provide a novel insights into the development of promising therapeutic strategy for T1DM-associated bone loss.

## Materials and methods

### Animal models

miR-214-3p knockout mice (C57B6J, female, 8 weeks old) were obtained from National Resource Center of Model Mice (NRCMM) and Model Animal Research Center of Nanjing University (MARC). C57B6J wild-type mice (female, 8 weeks old) were obtained from Beijing Vital River Laboratory Animal Technology Co., Ltd. Then, the mice were randomly divided into two groups: the diabetic group was intravenously injected with one dose of streptozotocin (STZ) solution (50 mg/kg, Sigma, St. Louis, MO, USA), meanwhile the control group was injected with equivalent volume of sterile normal saline. After 3 days of injection, the blood glucose concentrations were measured by a glucometer (OneTouch SureStep, Lifescan, Milpitas, CA). The mice were confirmed as diabetic when blood glucose concentrations were higher than 16.7 mM. All animals were housed under standard animal housing conditions (23−25 °C, 12−12 h light−dark cycle) and were given a regular diet. All the experimental procedures in this study were approved by Jinan University.

### Cell culture and clinical samples

For primary cell culture, bone marrow stromal cells (BMSCs) isolated from both the tibia and femur of mice were cultured in α-MEM (Invitrogen, New York, NY, USA) with 10% FBS (Gibco, Life Technologies, Grand Island, NY, USA), 1% penicillin and streptomycin (Gibco, Life Technologies, Grand Island, NY, USA) and 2 mM l-glutamine (Sigma-Aldrich, St.Louis,USA). To induce the osteogenic differentiation, osteogenic medium supplemented with 50 µg/ml ascorbic acid, 5 mM β-glycerophosphate and 10 nM dexamethasone was used (all from Sigma-Aldrich). The culture medium was replaced every 2–3 days. All cells were cultured in the cell incubator (37 °C, 5% CO_2_). To enhance or inhibit the expression of miR-214-3p, BMSCs were transfected with 200 µM AgomiR-214-3p or 200 µM AntagomiR-214-3p or their corresponding mutants or negative controls (Guangzhou Ribobio Co., Ltd). The medium was changed every 2 days. Besides, we conducted siRNA transfection assay to silence the expression of β-catenin. The siRNA sequence was used as follows: AGCUGAUAUUGAUGGACAG (sense) and CUGUCCAUCAAUAUCAGCU (antisense).

Bone specimens were collected from three healthy and T1DM osteoporosis patients at Peking University Third Hospital, Beijing, China. The tissues were collected with written patient informed consent and approved by the hospital.

### miRNA microarray and cluster analysis

To identify the differentially expressed miRNAs in STZ-induced diabetic mice, RNA was isolated from bone specimens of diabetic mice and normal mice. Then miRNA microarray was performed at CapitalBio Technology Ltd. The differentially expressed miRNAs were screened at a criteria of fold change >2.0 or <2.0 and a *p* value < 0.05. Heat maps representing the differentially expressed miRNAs were generated using Cluster 3.0.

### Dual energy X-ray absorptiometry and micro CT analysis

Representative dual-energy X-ray images of mouse femur were analyzed by dual-energy X-ray absorptiometry (DEXA; LUNAR Radiation, Madison, WI, United States). Thereafter, bone mineral density (BMD) of the intact femur from STZ-induced diabetic mice or the corresponding controls was analyzed.

For analysis of bone microstructure, the distal portions of the femur were scanned by a μCT scanner (µCT40, SCANCO MEDICAL, Switzerland) at a voxel size of 10 µm. The trabecular volume of interest (VOI) started at the region of the distal femur beginning at the growth plate and extending proximally along the femur diaphysis. The high-resolution images were constructed from 423 slices. Morphometric parameters including bone volume fraction (BV/TV), trabecular thickness (Tb.Th) and trabecular number (Tb.N) were quantified.

### Alizarin Red staining

To assess the osteogenic differentiation of BMSCs, calcium deposition and mineralized matrices were detected with Alizarin Red staining. After 21 days of in vitro osteogenesis, BMSCs were washed thrice with distilled H_2_O and then fixed in 70% ethanol for 1 h. After washing with distilled H_2_O, the cells were stained with 40 mM Alizarin Red S solution (Sigma-Aldrich, St.Louis,USA), pH = 4.0 for 15 min and washed thrice with distilled water. After washing five times with distilled H_2_O and cells were rinsed with 1× PBS and observed under phase contrast microscopy.

### Dual-luciferase reporter assay

To identify the target of miR-214-3p, we utilized PITA, miRanda and miRDB to search for the potential target genes, which identified binding site in the 3′-UTR of β-catenin for miR-214-3p. Thereafter, the 3′-UTR of β-catenin was PCR amplified from mouse genomic DNA and the amplicon was then cloned into the pGL3 empty vector ((Promega Biotech AB, NACKA, Sweden) for Luciferase reporter gene assay. The luciferase activity was analyzed 48 h after transfection using the Dual Luciferase Reporter Assay System ((Promega Biotech AB, NACKA, Sweden). Luminescent signals were quantified by luminometer (Glomax, Promega), and firefly luciferase activity was normalized to Renilla luciferase activity in each sample.

### Quantitative real-time PCR

Total RNA was extracted from bone tissues or BMSCs with TRIzol Reagent (Invitrogen, New York, NY, USA), according to the manufacturer’s protocol. After purification, 1 μg of total RNA was reverse transcribed into cDNA using a High-Capacity cDNA Reverse Transcription Kit (Applied Biosystems). SYBR Premix Ex Taq kit (Takara Biological Incorporated Company, Kyoto, Japan) was used for qRT-PCR analysis using the Bio-Rad iQ5 real-time PCR system (Bio-Rad, Hercules, CA, USA). GAPDH was served as an internal control for mRNAs. Fold changes in expression were calculated by the 2^−ΔΔCt^ method.

The miRNeasy Mini Kit (QIAGEN, USA) was used to extract miRNAs from bone specimens or BMSCs. The samples were lysed with a mixture of 700 µl QIAzol and 140 µl chloroform and centrifuged at 12,000 × *g*, 4 °C for 15 min. Thereafter, upper aqueous phase was transferred to an RNeasy Mini spin column in a 2 ml collection tube and mixed with 100% ethanol. The total RNA was collected after washing with 700 µl Buffer RWT and 500 µl Buffer RPE, then qRT-PCR analysis was performed. U6 was served as an internal control for miRNAs. Fold changes in expression were calculated by the 2^−ΔΔCt^ method.

### Western blot

Cells were harvested after washing with cold PBS and lysed in lysates containing protease inhibitor cocktail and phosphatase inhibitor cocktail (Pierce, IL, USA). Then protein concentration was measured using the BCA protein assay kit (Beyotime Biotechnology, Shanghai, China). Protein samples with equal protein content were subjected to SDS-PAGE and electro-transferred onto PVDF membranes. The membranes were blocked with 5% skimmed milk and incubated with a primary antibody at 4 °C overnight. Finally, membranes were probed with a horseradish peroxidase (HRP)-conjugated secondary antibodies and visualized using an enhanced chemiluminescence kit (Pierce, IL, USA). The following primary antibodies were employed: anti-β-catenin (Abcam, UK), anti-GAPDH (Abcam, UK).

### Statistical analyses

All statistical values were calculated using GraphPad Prism 6.0 (GraphPad Software, Inc., San Diego, CA, USA). Student’s *t* test was used to evaluate statistical differences between two groups, and one-way ANOVA was used for comparisons involving more than two groups. The level of statistical significance was set at *p* < 0.05 and *p* < 0.01.
